# Beyond ad hominem attacks: A typology of the discursive tactics used when objecting to news commentary on social media

**DOI:** 10.1371/journal.pone.0328550

**Published:** 2025-08-20

**Authors:** Ashley L. Shea, Aspen K. B. Omapang, Ji Yong Cho, Miryam Y. Ginsparg, Natalie N. Bazarova, Winice Hui, René F. Kizilcec, Chau Tong, Drew B. Margolin

**Affiliations:** 1 Department of Communication, Cornell University, Ithaca, New York, United States of America; 2 Department of Information Science, Cornell University, Ithaca, New York, United States of America; 3 School of Journalism, University of Missouri, Columbia, Missouri, United States of America; 4 Institute for Data Science and Informatics, University of Missouri, Columbia, Missouri, United States of America; Universidade Federal de Ouro Preto, BRAZIL

## Abstract

Social media platforms increasingly serve as the primary place where people participate in public conversations about news. In these conversations, ad hominem attacks are quite common. Such ad hominem attacks might be influenced by underlying cognitive or affective goals, such as to discredit a purveyor of falsified evidence or to signal social distance from a hateful provocateur. They may also be driven by a simple operative goal: to stop what was said. When ad hominem attacks are used to stop the comments of another person, we refer to it as a *discursive objection tactic.* In this paper, we explore the prevalence of ad hominem attacks and characteristics of other discursive tactics used by people when objecting to online news commentary. First, we conducted a content analysis of more than 6,500 comment replies to trending news videos on YouTube and Twitter and identified seven distinct discursive objection tactics. Second, we examined the frequency of each tactic’s occurrence from the 6,500 comment replies, as well as from a second sample of 2,004 replies. Our findings confirm that while ad hominem attacks are the most common discursive tactic used to object to news commentary, people also deploy a diversity of other discursive objection tactics. The resulting typology offers a comprehensive account of grassroots efforts which utilize deterrent speech, nonaccommodative communication and prosocial strategies.

## Introduction

Social media platforms increasingly serve as the primary place where people consume news and can participate in public conversations about it [1]. For example, more than 70% of U.S. adults consume news on social media [2,3] and roughly half have commented in response to the news that they consume [4,5]. As many legacy news sites enact paywalls and remove user-generated comment sections from their websites, social media platforms thus serve an important civic function of supporting public discourse [6]. Public conversations about news on social media are important for both commenters and onlookers. Indeed, a recent study found that people read comments despite not planning to engage directly when they want a quick synopsis of topical news issues [7].

Given their importance in social exchange, comment spaces are also a source of public concern. One worry relates to the potential for psychological or emotional harm to those who read comments. Comment sections can harbor racial hostility [8], sexual harassment [9] and calls for political violence [10], directly impacting the wellbeing of readers. Another concern is that the degradation of civil discourse will negatively influence how citizens engage in society [11]. In addition to content that can harm or marginalize specific individuals and entire identity-based groups, comment sections often contain incivility [12] and misinformation that can “spill over” to different social settings [13]. The presence of these forms of detrimental or “objectionable” content undermines civic participation and deliberation around important news topics. In simple terms, comment sections may be where people are going to discuss the news, but the substance and style of those discussions can lead to communication breakdown which impedes democratic discourse.

Perhaps the most general and widely known marker of communication breakdown is the use of shame to disparage the “face” or “reputation” of a speaker, thus discounting their point of view [14]. This tactic is called an *ad hominem* attack. Ad hominem attacks are problematic because they impede the process of resolving objections on the merit of argument alone [15]. Unfortunately, they are commonly used: for example, a recent analysis of comments left in a popular online debate forum revealed that nearly one-third of all comments there contained ad hominem attacks [16].

Though disconcerting, the prevalence of ad hominem attacks to deter objectionable content is understandable in social media conversations where moderation policies may be imperfect, unpredictable or incompatible with individual standards and expectations. In these spaces, individuals are navigating a “polluted” information environment [17] leading them to frequently experience moral outrage at the presence of objectionable content [18]. When individuals encounter this content, it can then provoke a sense of responsibility to engage in their own “expressive citizenship” [11]. Attacking others is a common tactic to try to silence immoral or dangerous content, performing the work of a digital vigilante that confronts a “corrupt person” who has not been appropriately sanctioned through official mechanisms [19].

This study investigates the distinct discursive tactics online users deploy to object to the content of another’s post. The idea of an “objection” is an analogy to the legal tactic of “objecting” to questions or statements so that they are removed from the “record”, the sanctioned evidence base for consideration in legal proceedings. Such tactics are an attempt to exert power to uphold—or shift—what the objector perceives as the correct dominant behavior or narrative. This may be because the statement is wrong according to a general standard or presumed universal norm—it promotes an immoral view (e.g., racism), or false information—or because it is simply not acceptable in that space (i.e., violates local/community norms). While such tactics may impose a penalty directly on the offender—such as when a user comments “*Enough with that racist garbage. I’m reporting you!”*—they can also indicate to others observing the conversation that the statement they object to does not belong. Our aim is to document a comprehensive set of these objection tactics to understand both their characteristics and their relative prevalence. This study is guided by the following research questions:

RQ1: What are the discursive objection tactics that people employ in online news comments, and how do these tactics align with strategies suggested by prior theory?

RQ2: What are the defining features of each discursive objection tactic?

RQ3: What are the relative frequencies of discursive objection tactics in direct comment replies in online news?

Prior work has focused on the who, rather than the how, of objecting [20]. Specifically, research has identified specific kinds of actors who try to deter objectionable online behavior in specific ways, such as vigilantes [19,21], digital defenders [22], hacktivists [23], digital upstanders [24] and cyber warriors [25]. In complement to this work, we examine the characteristics of discursive objection tactics used by people in online news commentary. First, we conducted a content analysis of 6,500 comment replies to trending news videos on YouTube and Twitter and identified seven distinct discursive objection tactics. Second, we examined the frequency of each tactic’s occurrence from the 6,500 comment replies, as well as from a second sample of 2,004 comment replies. Our findings confirm that while ad hominem attacks are the most common discursive objection tactic used in news commentary, people also deploy a diversity of other discursive objection tactics. The resulting typology provides an account of grassroots efforts which utilize deterrent speech, nonaccommodative communication and prosocial strategies.

### Understanding discursive objection tactics

Social media platforms try to promote and uphold their preferred normative standards for discourse through enforcement of platform policies and moderation [26]. However, some content that is perceived by users to be wrong might remain. This is in part because users can hold different understandings of normative expectations for these public conversations. For example, while “democratic” is often used as a shorthand for a set of values that guide discourse and deliberation [27], there are actually three distinct forms of democratic communicative norms that influence individual speech [28]. *Liberal individualist norms* promote free expression of individual ideas with little regard for others [29,30] while *communitarian norms* encourage interactions and collective action with like-minded members at the exclusion of outgroup members [29,31]. There are also democratic *deliberative norms* which embrace reciprocal heterogeneous idea exchange that is grounded in civility between people of diverse ideologies [29,32,33].

One way to understand these differences is to further explicate how democratic communicative norms relate to social norms more broadly. Social norms are the “frames of reference” which shape behavior within different social settings [34]. They include descriptive norms, or an individual’s perception of how the majority of others behave [35] and injunctive norms, or an individual’s perception of what should be done in a given context [36]. Sometimes, these norms are incongruent. For example, if an individual who favors democratic deliberative norms perceives that most people participating in a news discussion forum are “polluting” the forum with unproductive or problematic comments (descriptive), the individual might feel a mismatch between observed behavior and what they feel ought to be done to promote more productive democratic discourse (injunctive). This mismatch can result if different democratic communicative norms are coming into conflict. For example, one’s free expression can be incompatible with another’s expectation of deliberation and can produce intense negative reactions among interactants and observers alike [37]. In practice, this can then lead one individual to call out another for being uncivil and violating their expectation of deliberative norms, while the other may feel they are being illegitimately “silenced,” violating their individualist expectation for free expression [11,38].

The convergence of different individual normative preferences on platforms has thus created an environment where a consensus for decisions about what is inappropriate in a public discussion of the news is hard to reach. This, in turn, makes it very difficult for platforms to impose rules that are completely satisfactory to users. As a result, a culture of self-governance has emerged within public comment sections [39] where individuals engage in “lateral surveillance,” or the act of monitoring, judging and deciding on the appropriate response to moral infractions within the shared space [40]. In these contexts, it is common for individuals to perceive that another person not only disagrees with them but has said something “wrong” that should not be said, and thus should not be repeated. The content could be perceived wrong for many reasons, such as because it is inappropriate or offensive, because it is mean, or because it is viewed as dishonest or false.

Despite the underlying normative complexity that generates many of these conflicts, users often experience these statements as violations that require redress, such as through upstanding [41] or factual correction [42]. In other words, when this violating content appears, some people choose to “object” to it because they perceive it to be the kind of thing that should not be said, and they feel compelled to say something. We call this a *discursive objection tactic,* which we define as an attempt to restrict the ways in which others can speak. We use the term *discursive* to denote two important properties. First, the term reflects the assertion of relational power amidst competing ideologies [43] which the affordances of digital platforms—particularly commenting, as we argue- enable. Second, discursive tactics are broader than rhetorical tactics [44,45]. While they can be employed to persuade, they can also be employed to manipulate, to coerce or to intimidate, as is the case when someone comments “you want to try restricting my freedom? bet I can find your address in 5 and have someone at your door in TWENTY. You can’t restrict ANYTHING”. Discursive objection tactics emerge where assertion of force and ideology collide [45] through “centrifugal and centripetal forces” [44], including norms, values and the technological affordances available. Discursive objection tactics emerge in the space between non-textual forms of objection also afforded by social media platforms (such as flagging, downvoting and “blocking”) and the textual forms of objection that presume a persuasive intent and technique of the communicator. Broadly and for the purpose of our project, “discursive” means “anything that could be said that might act as a deterrent or inducement to stop the bad behavior.” A person steps in and employs a response tactic to exert communicative control over content within their social environments [46]. This “stop command” is a way of signaling to the original speaker, as well as to any others who are observing, that this kind of communication will not be tolerated. These stop commands often include a verb (e.g., “You shouldn’t say that”) but may also rely on nouns that implicitly call on a commenter to stop (“Liar!”). A stop command is characterized not by a particular form but by its implication—that the person saying this should not say it. A single discursive objection tactic can serve a dual purpose: Beyond the short-term goal of stopping the immediate behavior, it can also aim to achieve long-term goals, such as to prevent similar content in the future or to promote different norms within the space. Calling someone a liar, for example, can have an immediate impact on the recipient while also achieving a lasting chilling effect that signals to others that they too will be labeled similarly if they repeat the comment [47].

To understand how objection tactics might manifest in people’s comments, we review literature on three distinct types of deterrent speech with theoretical foundations that will help us conceptualize the results of our analysis.

### Mechanisms of deterrent speech

Deterrent speech is a statement intended to thwart and prevent unwanted behavior [48]. Deterrent speech emerges from deterrence theory in the criminal justice literature and draws on deontic principles to uphold and preserve what is considered permissible and obligatory by warning that impermissible and disobedient behavior will be sanctioned [49]. To date, deterrent speech has been examined in digital and offline contexts as a strategic and performative practice of key political or authoritative actors [50] to stop and/or prevent what is perceived to be problematic behavior from adversaries. Deterrent speech uses threats, friction, or internalized deterrence to stop unwanted behavior [49,51,52].

### Threats

Threats are characterized by a “coercive” attempt to motivate behavior change by imposing sanction or harm to one’s reputation or safety [53]. On social media, threats can manifest as ad hominems which attack the characteristics of a person and threaten their social image, regardless of whether such characteristics are relevant to the topic being discussed [14]. In addition to punishing the recipient, ad hominems also threaten the onlooker by suggesting that similar offenses will be met with a similar response. Ad hominem attacks have been referred to as “strategic maneuvers” [15] because they are driven by goals of the attacker, such as wanting to discredit a purveyor of falsified evidence [54] or signaling social distance from a member of another party because they appear unreliable, suspicious or inadequately informed [55,56]. Threats of blackmail or doxxing are another type of threat which risks the safety of the “face” by promising to reveal sensitive and harmful information about a person if their behavior does not change [57]. Threats can also be physical when they directly state or imply that there will be violence in response to a statement [58], as comments to #stopthesteal have exhibited [59].

### Friction

Another way that people might object in public discourse is by disturbing or agitating the speech flow with friction. A useful theory for understanding the mechanisms which promote and hinder speech flow is Communication Accommodation Theory (CAT) in the interpersonal communication literature [60]. According to CAT, speech friction is a form of *nonaccommodative* speech which fails, intentionally or unknowingly, to promote flow by emphasizing differences with a communication partner which are inferior or intolerable [61,62]. On social media, friction can be observed as “trolling” comments which repeatedly disrupt and impede conversations through provocative and often non-topical interjections [63]. This friction *can* veer towards harassment and include ad hominem attacks, but it does not have to: it can also manifest as an interfering annoyance, such as repeated comment replies using a string of emojis designed to mock the conversation (Shi & Lam, 2016). Friction can also include discursive deflection in which replies impede the progression of a conversation by changing what is being discussed [64]. Friction also includes microaggressions such as implicit stereotypical language [65] or explicit requests to stop doing what they are doing [66], which can impact one’s sense of belonging in the conversation and cause enough friction to make a person want to leave [67]. An example of this is telling someone that they or their words “don’t belong here” [66].

### Internalized deterrence

Internalized deterrence is a third type of deterrent speech and works without threats or friction [68]. Instead, compliance with a norm is achieved through speech which activates an individual’s sense of internalized or institutionalized rules, including pro-social deontic norms of what is considered right and wrong [69]. This can be done through moral reasoning [70] or social norm nudging [71]. Moral reasoning plays on emotion and logic by stating a presumption of what is in the best interest of the greatest number of people [72], while social norm nudging exposes users to information about “typical” moral behaviors that are also desirable [73]. Perhaps the most widely internalized norm is honesty [74]. This norm can be evoked by saying “let’s try to be as honest as we can” to elicit a commitment to the truth while showing that the particular statement drawing an objection does not match the moral standard [75].

Together, these types of deterrent speech suggest plausible scenarios that may emerge in news comments. We set out to identify comment sections where objections might be visible and prevalent.

### News comments as fertile ground for objections

Ample research shows that online interactions occur in echo chambers among like-minded people [76], reducing the potential for direct interaction between people who share different views. However, while echo chambers [77] and information cocoons [78] can insulate people from heterogeneous information, recent studies have demonstrated that *cross-talk*—conversations between individuals with different views, particularly on political or other deeply held issues, is also common in the news comments on social media. For example, in a recent study of cross-partisan discussions between active liberal and conservative users (e.g., people that have left at least 10 comments) on YouTube, researchers found a surprising amount of cross-talk: 69% of active users posted at least once on both left-leaning and right-leaning YouTube channels [79]. In another study, researchers found that social media “crossovers”—people that leave comments on ideologically opposing news forums—are pulled to the opposing news forums both out of curiosity as well as the more morally motivated desire to “share the truth.” [80]. In these situations, comment sections can serve as an “battleground” where opponents try to exert power and control over one another [81]. Such battlegrounds are thus likely hotbeds of discursive conflict.

Cross-talk also occurs when people with different views and values are pulled into the same comment sections when exposed to platform-level daily “trending” lists on YouTube (and on Twitter, “what’s happening” trending lists) that promote news stories that are most viewed and shared [82]. When people consume content from “trending” lists they might be exposed to a broader array of news than they would be based on their own political or ideological leanings [83] and it follows that user-generated comments beneath the content should reflect this inter-ideological mingling, with some people trying to advance partisan agendas [84] and others attempting to silence others. This is precisely where we should expect to observe distinct objection tactics.

YouTube has been described as a site of “intense collective sense-making” where YouTube’s comments can function as an “under-regulated epistemic space” [85]. On YouTube, the default setting is to display comments according to engagement, where parent comments with the most likes and/or replies are presented at the top. While the option exists to display comments according to what comment is most recent, the default setting means that the most “popular” is often seen instead. On Twitter too, comments are made more visible by platformed publics that perform “affective expressions” through technological affordances, such as retweeting and replying [86]. On Twitter, the default setting in the API is to return replies chronologically (although the option to sort by engagement is also available), while the user website and interface prioritizes content deemed most relevant and engaging. This kind of scoring of our communication influences what gets seen, and thus what generates a reply, and perhaps even, what kind of reply is given [87].

## Materials and methods

### Study design

**Fig 1:**
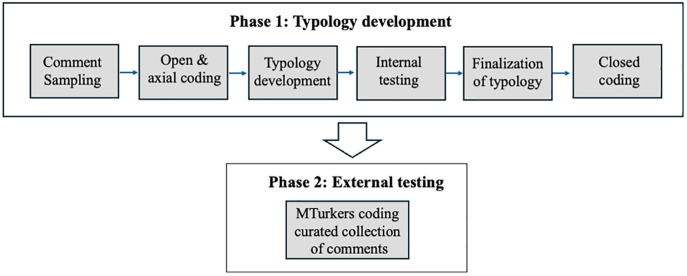
Process diagram of the typology development and testing phases.

This study investigates the discursive objection tactics employed in news comments. To answer RQ1—in which we seek to identify the distinct tactics—and then RQ2—in which we seek to identify their defining features—we employ a two-phase approach to first develop and then validate a typology of discursive objection tactics derived from comments on social media platforms (Twitter and YouTube) where cross-talk occurs. In the first phase, we used a six-step process informed by content analysis [88] and collaborative thematic analysis [89] where we conducted 1) comment sampling, 2) open & axial coding, 3) preliminary typology development; 4) internal testing, discussion and reconciliation; 5) finalization of the typology and 6) closed coding. In the second phase, we sought to determine whether the codebook could be readily applied at scale by external coders. This involved investigating whether the distinct tactics and their defining features were discernable enough to be learned and applied by external coders [90] unfamiliar with the initial study conditions. Here, we trained crowd workers from Amazon’s Mechanical Turk (“MTurkers”) to apply the coding scheme to a collection of curated comments previously sampled and coded in the first phase and measured their accuracy against the “ground truth” established by our research team. The components of phase 1 and phase 2 are illustrated below (Fig 1).

### Comment sampling

In line with research showing substantial cross-talk across ideological news outlets [79] and trending news content [91], we sought comments from popular and timely news videos. In total, our data consisted of two samples. The first sample included direct replies drawn from YouTube and Twitter between August 31, 2021 and October 22, 2021, prior to Twitter’s ownership change [92]. The collection of data complied with the terms and conditions for both YouTube and Twitter. These platforms were selected because at the time the data samples were collected, the platforms were among the most popular social media platforms for consuming news [2]. Additionally, YouTube’s popularity across various age, gender, and racial groups [3] and Twitter’s large share of news-focused users [93] made each attractive options. To identify popular news topics generating interest and discussion beyond a single platform, we looked at Google’s trending searches API as well as the subreddit “r/changemyview” (r/CMV) threaded discussions, a forum examined in prior research [94]. Both are popular mechanisms used to identify “buzzworthy” topics online, with the former showcasing what topics are generating search exploration [95] and the latter reflecting what topics are generating discussion [88,89,96]. It was important that we acquire our list of buzzworthy topics from two sources rather than relying on one because some topics could conceivably be unique to one platform only. Thus, to ensure broad appeal that transcended platform-specific trends, we cross-verified the topics list [97]. In total, fourteen news topics were represented across both sources between August 31, 2021 and October 22, 2021. With our news topics identified, we then turned to YouTube and Twitter for comment sampling, searching the first 10 of 14 topics on YouTube and the remaining four topics on Twitter and selecting the video per topic with the most views. More videos were sampled on YouTube (i.e.,10) than Twitter (i.e.,4) because despite Twitter’s large share of news-focused users, YouTube has a significantly larger share overall of Americans that use it regularly (81%) compared to Twitter (23%) [3].

A total of 14 videos were combined to comprise our first sample, with the top-ranked (based on each platform’s engagement metrics) 100 parent comments and their reply chains scraped from each video. Altogether 7,500 direct replies (e.g., replies directed at another user) were amassed; 6,500 would be used for content analysis, with 1,000 set aside for inter-coder reliability checks. Table 1 lists news topics and video sources for this first sample, as well as the share of direct replies for each video within Sample 1.

**Table 1:** News issues in sample 1.

News topic	Date collected	Platform	Video source	Direct replies (percentage of sample)
Anti-Asian hate crimes up	8/31/2021	YouTube	James Corden Show	412 (5.5)
FDA approval of Pfizer	8/31/2021	YouTube	MSNBC	415 (5.5)
Kabul airport evacuation	8/31/2021	YouTube	The Hill	606 (8.1)
Hurricane Ida	8/31/2021	YouTube	NBC News	804 (10.7)
Debt ceiling	10/5/2021	YouTube	Bloomberg TV	718 (9.6)
California oil spill	10/5/2021	YouTube	ABC News	203 (2.7)
Merck Covid pill	10/8/2021	YouTube	CNN	828 (11.0)
Mass shooting in MN	10/12/2021	YouTube	NBC News	780 (10.4)
Vaccine protest	10/12/2021	YouTube	MSNBC	636 (8.5)
Teaching about Holocaust	10/19/2021	YouTube	CNN	513 (6.8)
Critical Race Theory	10/22/2021	Twitter	The View	427 (5.7)
BLM protests	10/22/2021	Twitter	Vox	372 (5.0)
Math teacher offends	10/22/2021	Twitter	NBC	349 (4.7)
Kabul chaos	10/22/2021	Twitter	CNN	437 (5.8)

News topics in the first sample reflect concerns of both the public and private sphere. Prior research has found that of the U.S. adults who comment on news online, women are less likely than men to comment on “public sphere” issues like politics and foreign affairs but more likely than men to comment on “private sphere” issues including stories about parenting and health [98]. In sample 1, we have both types, including the evacuation of U.S. troops from the Kabul airport (public sphere) and the Merck Covid pill for treating Covid symptoms (private sphere).

The second sample was drawn 8 months following the first sample. Whereas for the first sample we were interested in conversations where cross-talk and objections were likely most prevalent for our content analysis, for sample 2 we simply wanted the largest sample to assess relative frequency of the objection tactics which comprise the typology. Thus, we selected the top 10 videos with the greatest number of total comments under the “US News” category on the official and heavily viewed CNN YouTube channel on August 16, 2022. We then randomly selected 10% of the direct replies to top-level parent comments left within the first 3 hours of each video posting. This produced a sample of 2,004 direct replies, which we analyzed. Table 2 lists news topics and video sources for this second sample, as well as the share of direct replies for each video within sample 2.

**Table 2:** News issues in sample 2.

News topic	Date collected	Platform	Video source	Direct replies (percentage of sample)
Will Smith punch	8/16/2022	YouTube	CNN	263 (13.1)
Anti-abortion activist	8/16/2022	YouTube	CNN	68 (3.4)
Putin speech	8/16/2022	YouTube	CNN	129 (6.4)
Capitol rioter	8/16/2022	YouTube	CNN	238 (12.0)
Texas mass shooting	8/16/2022	YouTube	CNN	269 (13.4)
Will Smith at Oscars	8/16/2022	YouTube	CNN	194 (9.7)
Brittney Griner’s sentence	8/16/2022	YouTube	CNN	403 (20.1)
Buffalo shooting suspect	8/16/2022	YouTube	CNN	183 (9.1)
Joe Rogan misinformation	8/16/2022	YouTube	CNN	92 (4.6)
Warren’s response after Roe v. Wade ruling	8/16/2022	YouTube	CNN	165 (8.2)

### Data analysis

#### Open & axial coding.

With sample one, we engaged in open and axial coding. The purpose of open and axial coding was to: 1) identify categories that objection tactics could be sorted by, 2) distinguish between categories, 3) define each category clearly, and 4) ensure that categories were comprehensive, catching every tactic we encountered. In our coding, we assessed direct comment replies to other users only, not parent comments left in response to the original video. This ensured that we focused on social interactions between two people, where users object to what another person said. It also ensured that coders were not influenced in their assessment of whether the objection was “reasonable” or “justified” with regard to the parent comment. A team of four graduate students distributed 500 comments among themselves, with each open coding 125 comments. Throughout open coding, they met frequently to discuss potential categories and labels that emerged from independent sorting and labeling. Usernames were removed from direct replies and the coder focused purely on the logic of the objection—the mechanism by which the objector created an incentive for this speech to stop. All direct comment replies that contained examples of ad hominem attacks, nonaccommodative communication (including “talking down” or disparaging while emphasizing social identity differences and explicit calls to stop talking or leave) and deterrent speech (including explicit and implicit threats, and invocation of deontic principles) were set aside in a singular corpus of objections. In short, any comment that included what a coder considered a “stop command” was flagged.

### Typology development

Following the preliminary trial open coding in which categories of objection tactics emerged, two of the original four graduate students sought to apply the categories to 500 additional comment replies previously set aside that remained unanalyzed, with each coder independently coding and selecting the first code relevant to every comment. This was to ensure that emergent categories were sufficiently comprehensive as well as distinct and well-defined. For example, in the comment “hey racist, get out of here”, the coder should assign it with “ad hominem attack” because the attack appeared before the admonishment to leave. All objection tactics were classified in at least one of the emergent categories, with non-objection comments not classified.

### Internal testing and finalization of typology

Following acceptable reliability of a Krippendorf’s alpha of 0.60 with 88% agreement between the two coders, the typology of the objection tactics was finalized. To answer RQ1—in which we sought to identify the distinct tactics—and then RQ2—in which we sought to identify their defining features—we present the tactic labels, definitions, and an exemplar comment for each are shown in Table 3.

**Table 3:** Typology of objection discursive tactics.

Objection tactic	Definition	Exemplar comment
Moral corruption	Replies stating or implying that the comment is morally corrupt because it violates some principle or norm, or suggests that there is a morally superior way of stating the point.	@User If you are a veteran then you know you can’t say that, be proud and a soldier
Logical disqualification	Replies that state or imply that the comment is false and cannot stand because facts or logic prove otherwise.	@User NO,Raising the debt ceiling pays bills mostly earmarked and spent during the Trump years. You should study up before posting what you don’t understand.
Physical threat	Replies that intimidate a person by directly threatening or implying violence toward them, or their in-group.	@User Anybody who attempts to ban/outlaw AR-15’s or AR-10’s from We the People deserves death by the most painful means possible.
Ad hominem	Replies that use accusatory labels to attack or smear the reputation of the person that they are responding to.	@User Quacks like a RACIST republican, you get called a RACIST republican
Content threat	Replies that use accusatory labels to attack the content of a comment and dismiss it outright.	@User FAKE NEWS!!!!!
Self control	Comments that directly state or signal self-exit from the conversation or platform.	@User I’m not talking to you anymore because it’s.. just exhausting.. literal EVERYTHING you said is a lie. And you’re biased and delusional... We’re done.
Space control	Comments that direct a person to remove themselves or their comment from the conversation.	@User you make no sense. Go back to the kiddie table and let the adults talk. I’m sorry if the truth makes you so butthurt. Run along now. You’re dismissed.

The first two tactics—moral corruption and logical disqualification—share similarities with deterrent speech, particularly with their emphasis on deontic principles of right and wrong. However, both tactics tend to lack a punishing element and instead utilize prosocial language to uphold respectful and supportive dialog. For example, moral corruption can present as “BE. BETTER.” while logical disqualification can present as “Wrong! False. Impossible! Try again.” The next tactic—physical threat—aligns with the form of deterrent speech known as deterrent *threats*. Physical threats explicitly or implicitly threaten violence at the target of the objection and utilize words which connote violence (“you better join a militia because the time for war is coming”). The next two tactics are ad hominem attacks and content threat. While ad hominems attack the “face” or *character* of a person and content threats target the *content*, both possess elements of implicit deterrent threats, particularly if receipt of such a branding or label punishes the offender and deters repeat offense from the offender, or from other onlookers. Together, ad hominems, content threats, and physical threats always use nonaccommodative language to accentuate salient or perceived differences between communication partners.

The remaining two tactics—self control and space control—may incorporate elements of deterrence by citing deontic principles of what is good and bad to say in the space, but primarily utilize nonaccommodative speech to stop interactions and illustrate communicative boundaries by signaling division between in-group/out-group membership and belonging. In self control, the stop mechanism is for the offended individual to leave the conversation or space. That is, rather than attempting to stop the behavior from occurring in the future, they state that they are withdrawing so that they will not, themselves, witness it anymore (“I’m leaving”). In space control, the mechanism is similar but the locus of action is reversed. Here, the offender is told to leave the space, not so that they stop doing the objectionable behavior, but so that they stop doing it here, in this space, where the objector is present (“You don’t belong here, please leave”).

All seven tactics appear individually, but can also appear in tandem with one another. Some threatening tactics—particularly ad hominem attacks—also utilize terminology that might be intended to skirt algorithmic detection and removal (for example, “you YT devils” = “you white devils”). Logical disqualification and moral corruption tend to involve longer comments and utilize descriptive words related to logic (“I need to reason with you…”) or morals (“please check your moral compass before repeating that”), respectively. Content threats are often short retorts (“lie!”) and ad hominem attacks are short brandings (“racist”). Physical threats are to the point, as are space control and self control which tend to quickly communicate a directive or decision and may or may not include corresponding rationale (“you’re a waste of my time. I’m leaving”).

### Closed coding of sample 1 and sample 2

Following finalization of the typology, the remaining 6,500 of the 7,500 comments from sample 1 were distributed and close coded by the same two coders where reliability was previously established. They each reviewed and coded direct reply comments in batches of 800.

Comments in sample 2 were also coded using the same typology of discursive objection tactics. To expedite the manual process, a new coder joined the two graduate students that previously worked together. Thus to confirm uniformity in application of the typology again, the 3-person coding team independently coded 200 identical replies from the second sample of 2,004 comment replies. Following independent coding, the team reviewed discrepancies and clarified areas of divergence through three hours of reconciliation. The results of this testing were very good, with a Krippendorf’s alpha of 0.74 and 95% agreement. Our alpha of 0.74 was a better result than the alpha of 0.60 calculated previously and is indicative of the team’s growing familiarity with the typology and features of each category, as well as the importance of extensive training and discussion with example comments. Following the acceptable reliability, the team moved ahead to code the remaining 1,804 replies pulled for Sample 2, with each receiving 601 or 602 unique comment replies pulled from all 10 videos. The analysis of direct replies complied with the terms and conditions for both platforms.

### External testing of typology

After establishing internal validity among our highly trained internal coders, we turned to external coders, namely, crowd workers on Amazon’s Mechanical Turk. We used MTurkers for four reasons. First and foremost, it was an exercise to establish external validity [90]. Specifically, we needed to determine whether individuals without access to our research team’s history and perspective regarding objection tactics could learn to understand and identify them within real social media comments. In particular, it was possible that the codes were consistently applicable only to individuals in our team with knowledge of their initial construction with refined ability to detect their patterns. Thus, turning to MTurkers ensured that our findings were not limited to specific participants. Doing so also confirmed that each tactic was clear, well-defined and explainable with contextual fidelity [99]. Second and related, for practical purposes we wanted to determine whether online training modules were adequate for providing instruction on recognizing and classifying objection tactics, thereby opening pathways for scalable instructional and interventional opportunities in the future. Translating our research for practical applications including in education is an important long-term goal for the authors. Third, since we did not calculate intercoder reliability between graduate coders at the tactic level but rather on the sample level, use of MTurker coding enabled assessment at the tactic level to discern whether MTurkers fared better on some categories of tactics. Finally, MTurk afforded scalability in a way that graduate student coders could not.

Adult survey respondents, 18 years or older, were recruited between July 6, 2022 and November 3, 2022 from the crowdsourcing website Amazon Mechanical Turk using the MTurk Toolkit on CloudResearch [100]. CloudResearch is an online study management tool that provides infrastructure to crowdsource research tasks through externally-hosted surveys like Qualtrics. Past studies on Amazon Mechanical Turk have raised concerns about the quality of data collected from online workers [101], but recent studies demonstrate that screening out participants with low approval ratings and completion rates are sufficient mechanisms for ensuring high quality data [102,103]. We restricted recruitment to MTurkers living in the U.S. with English language competency because our comment sample consisted of replies to U.S. media content in English.

Participants were randomly assigned to one of the seven trainings. Each training consisted of written informed consent, a tutorial to define an objection tactic and illustrate its use in practice (see Appendix), a six-item quality-check quiz (Appendix), and for those that correctly answered at least 5 of 6 questions on the quiz, a test of 8 additional questions pre-coded by our research team. In this test, MTurkers were provided with random comment replies from the pre-coded sample and answered either yes or no to whether each comment utilized the learned objection tactic. In other words, because MTurkers were only trained on one tactic, rather than asking the comment to be categorized into one of the seven tactics, we asked them to simply say if the comment contained characteristics of their learned tactic (yes/no). This decision to only train MTurkers on one tactic was made taking into account the average task length recommendations and attention span for crowdworkers. This decision was also informed by a similar research design by Liu & Mcleod [104] in which different counter-framing approaches (alternative framing or direct challenging) were treated as separate conditions. Respondents were paid $2.20 for the tutorial and quiz, plus $2.20 additional if they completed all questions on the test. At least 3 MTurkers rated each comment.

### Ethical considerations

Ethics approval was sought from Cornell University Institutional Review Board for Human Participants; the study was deemed not to require ethics approval (IRB # 2104010305). The only involvement of human subjects was through online quizzes and the observation of public comments. For the online quizzes, each accompanying training consisted of written informed consent using Qualtrics that MTurk participants completed. The comments downloaded from videos on YouTube and Twitter were anonymized.

## Results

### MTurk results

A total of 371 MTurkers were recruited and completed the quiz. The mean quiz pass rate ranged from 32.00% to 82.35% across tactics, indicative of the range of difficulty that accompanies tactics that vary in their visibility and distinction, but also the range of quality among survey respondents. Of the 234 (63.07%) that passed the quiz and proceeded to the test, the scores improved substantially, with the average mean test scores for respondents ranging from 86% to 94%. Among test takers within each condition, the average agreement ranged from 89% to 98%, with at least 3 ratings per comment (1 rating per person). Results of Mturker coding are presented in Table 4.

**Table 4:** Results of MTurkers coding a curated collection of comments.

Objection tactic	Participants recruited	Avg. quiz score^a^	Quiz pass rate	Participants passed quiz	Accuracy^b^	Alpha^c^
Content threat	50	4.20	32.00%	16	94%	0.76
Logical disqualification	55	4.25	34.55%	19	86%	0.63
Moral corruption	52	4.48	55.77%	29	86%	0.56
Self control	56	5.14	78.57%	44	89%	0.72
Space control	48	4.98	77.08%	37	90%	0.78
Ad hominem	51	5.20	82.35%	42	88%	0.65
Physical threat	59	5.07	79.66%	47	87%	0.71

^a^Average number of correct answers on 6 quality check quiz questions.

^b^Percentage of correct answers on 10 test questions among the participants who passed the quiz.

^c^Krippendorf’s alpha.

In using crowd workers, we found that novices could learn and identify objection tactics, and for those that passed the quality check quiz, the online training module provided adequate instruction that yielded relatively high test scores. These findings indicate that the codebook and the definitions within it are sufficiently clear to be applied by external subjects—namely, participants not involved in the study design. This finding satisfied our second phase of our study in which we sought to confirm that the codebook was generalizable and whether individuals could, in principle, be educated to recognize these differences as part of a training program. However, our results also showed that scaling application of this codebook will be challenging. Specifically, while for those that do learn these tactics (i.e., who pass the quiz), test accuracy was on par with or, in the case of content threats, better than the test scores observed for other tactics, results indicated that finding and training capable subjects was difficult. The discrepancy in quiz pass rates across tactics- particularly with content threats and logical disqualification each seeing less than 35% of participants recruited passing the quality check quiz- suggests that these tactics may be initially hard to learn. Also, the high rate of “dropouts” were costly. Nearly 40% of those recruited and that commenced the training failed to meet the standards (all were compensated for their participation in the training and quiz). This indicates that while the codebook identifies useful, meaningful constructs that individuals can learn to distinguish, scaling its use through crowd-workers would be expensive.

### Assessing tactic frequency across news videos

In total, 566 comments from sample one and 157 comments from sample two contained objections with at least one of our tactics present. The proportion of objections within Sample 2 (7.8%) was consistent with the proportion of objections from Sample 1 (8.7%). To answer RQ3—in which we sought determine the relative frequencies of discursive objection tactics in direct comment replies in online news—we report the frequency of objection tactics from both samples in Table 5 below.

**Table 5:** Frequency of objection tactics across sample 1 and sample 2.

Objection tactic	Sample 1: Youtube and Twitter (566 objections from 6500 replies; 8.7%)	Sample 2: Top 10 Youtube news videos (157 objections from 2004 replies; 7.8%)
Ad hominem attack	240 (42.4%)	71 (45.2%)
Logical disqualification	130 (23.0%)	26 (16.6%)
Content threat	67 (11.8%)	17 (10.8%)
Moral corruption	47 (8.3%)	32 (20.4%)
Space control	38 (5.0%)	9 (5.7%)
Self control	26 (4.6%)	1 (<0.1%)
Physical threat	18 (3.2%)	1 (<0.1%)

From our analysis, we find that ad hominem attacks are the most frequently used tactic, comprising between 42.4% and 45.2% of objections across both samples. Physical threats and self control tactics are the least common across samples. Logical disqualification and moral corruption were also common, with logical disqualification the second most used tactic in the first sample, and moral corruption the second most used tactic in the second sample.

In Table 6, we report the frequency of objection tactics across different videos for Sample 2. While the volume of comments in the first three hours of posting varies across videos, we found at least ten examples of objection tactics within each video’s comment sample. One video had as many as 32 objections.

**Table 6:** Frequency of objection tactics by video from sample 2.

Video	Comments coded	Total Objections	Ad hominem	Logical disqual- ification	Content threat	Moral corruption	Self control	Space control	Physical threat
Sara Sidner: I have three words for Will Smith	263	16	6 (37.5%)	1 (6.3%)	3 (18.8%)	4 (25.0%)	0 (0%)	2 (12.5%)	0 (0%)
CNN anchor challenges anti-abortion activist	68	10	6 (60.0%)	2 (20.0%)	0 (0%)	2 (20.0%)	0 (0%)	0 (0%)	0 (0%)
CNN reporter identifies strange moment in new Putin speech	129	14	8 (57.1%)	4 (28.6%)	1 (7.1%)	1 (7.1%)	0 (0%)	0 (0%)	0 (0%)
Son who turned Capitol rioter in reacts to father’s sentence	238	12	7 (58.3%)	0 (0%)	1 (8.3%)	2 (16.7%)	0 (0%)	2 (16.7%)	0 (0%)
Mass shooting at Texas elementary school kills at least 15	269	32	8 (25.0%)	5 (15.6%)	6 (18.8%)	12 (37.5%)	0 (0%)	0 (0%)	1 (3.1%)
Academy ‘strongly considered’ removing Will Smith from Oscars, source	194	13	7 (53.8%)	2 (15.4%)	0 (0%)	3 (23.0%)	0 (0%)	1 (7.7%)	0 (0%)
See Brittney Griner’s reaction as sentence is read	403	13	5 (38.5%)	3 (23.1%)	3 (23.1%)	1 (7.7%)	0 (0%)	1 (7.7%)	0 (0%)
What we know about the Buffalo shooting suspect	183	25	11 (44.0%)	5 (20.0%)	1 (4.0%)	5 (20.0%)	0 (0%)	3 (12.0%)	0 (0%)
Guest corrects Joe Rogan live on his own show. See his reaction	92	12	7 (58.3%)	2 (16.7%)	2 (16.7%)	0 (0%)	1 (8.3%)	0 (0%)	0 (0%)
Spitting mad’: See Warren’s furious response after Roe v. Wade ruling	165	10	6 (60.0%)	2 (20%)	0 (0%)	2 (20%)	0 (0%)	0 (0%)	0 (0%)

In all but one of the videos in sample 2, ad hominem attacks are the most frequently used category, comprising as much as 60% of all objection tactics. Moral corruption was the second most used tactic, accounting for at least 20% of objections in six of the ten videos. Physical threat and self control are the least used tactics, each appearing once.

## Conclusion & discussion

In this study, computational methods and content analysis were combined to investigate the characteristics and frequency of distinct objection tactics in news comments online. Our approach for comment coding yielded seven distinct objection tactics, representing a range that utilize deterrent speech, nonaccommodative communication, and prosocial strategies. Sample 1 and sample 2 were both analyzed to assess the frequency of objection tactics, and our results shed light on the variety of tactics that people employ in social spaces where people across the political and ideological spectrum converge.

Our first finding is that most objection tactics conform to the logics prescribed by theory. Three of the tactics we observed, including the most frequent (ad hominem attack) reflect the deterrent tactic of threat. However, consistent with internalized deterrence which suggests that individuals can draw on internalized moral rules to regulate behavior [68], we also found that deterrent speech can be free from threats and instead use prosocial attempts to frame desired norms in helpful speech. In particular, moral corruption and logical disqualification tended to be the second most used tactic in news comments (after ad hominem attack), and these tactics embraced deontic principles like obligation and duty when objecting to content on the basis of moral corruption or logical disqualification. We also saw tactics which incorporated friction. In self-control and space-control, individuals are explicit in their desire not to accommodate. In doing so, they also demonstrate a recognition that social norms are not necessarily universal, but require the consensus of participants. One way to object is to control exposure, rather than behavior, by explicitly stating who should stay in and who should leave the conversation space.

Beyond their alignment with theoretical principles, our work shows that people deploy a diversity of discursive tactics to object and can be used in different ways, such as in combination with other tactics. The diversity of discursive tactics underscores the importance of understanding the many factors that might shape objections. For example, there may be individual factors that encourage the same person to object in a consistent way across instances of offense. But there may also be contextual factors that encourage certain types of objections. From our sample, tactic type seems to depend on the nature of the video being discussed. Some videos, in particular videos that discuss mass shootings, seem to elicit more comments which utilize the moral corruption tactic. Perhaps we see these tactics here because of concerns about escalation when discussing violent content, or because users feel there is a consensus on moral norms (i.e., that violence is wrong). These are just some possibilities pointing to the fact that exploration into this is needed.

Despite the diversity of tactics available to people, ad hominem attacks are consistently used most frequently, and indeed by a significant margin. Affirming a general sense that social media is full of “shaming,” “flaming” and harassment, and consistent with arguments that moral behavior is instructed and enforced through sanction [105], we observed many instances of name-calling and “othering.” This category most closely resembles the idea of moral outrage or “moralistic punishment,” a behavior which can help hold bad actors accountable but can also exacerbate social conflict by dehumanizing others and escalating destructive feuds [18]. It seems, from our sample, that when individuals object to something and choose to intervene, they most often choose this option.

The frequency of this tactic raises the question of how often flaming, vitriolic interactions online begin this way, with someone smearing and undermining another person for what they perceive to be an offense. It is common to attribute online conflict to bad actors, but it is possible that people do this because they think this is the only way to stop what they perceive to be wrong speech. This also aligns with conceptions of digital vigilantes that choose deviant attacks (Trottier, 2020) in the name of re-establishing civil order [106].

In using crowd workers (MTurkers), we found that novices could learn and identify the objection tactics which emerge in complex spaces where ideologies and normative expectations converge. For those that passed the quality check quiz, the online training module provided adequate instruction that yielded relatively high test scores. This matters because the ability to recognize objection tactics confirms their unique characteristics, while the discrepancy in quiz pass rates across tactics suggests that despite objections being an important part of news commentary, some tactics may be difficult to detect and be initially hard to learn, particularly for crowd-workers engaging in the task on a single and temporary basis.

To our knowledge, this is the first study to conduct a grassroots accounting and analysis of the objection tactics employed in online comment sections. The paper is an important addition to the literature on online discourse and discursive conflict, more specifically. Existing literature that examines respondent behavior in the face of objectionable content (e.g., fact-checkers confronting incorrect information) often omit full accounting of the ways in which value-laden communication unfolds at the nexus of conflict, thereby lacking nuanced appreciation for the variety of organic forms of discursive resistance. Identifying strategies that commenters already employ to address objectionable content is the first step in better understanding their efficacy and ultimately identifying solutions to promote more prosocial discourse in the news comments on social media.

Indeed, our ongoing studies have examined the efficacy of different objection tactics on audiences’ interpretations and approval of them [107]. Such exploration can inform the creation of scalable online learning modules to train social media users on how to be effective objectors when encountering a discursive offense in social media. Future work also aims to explore the performance of different large language models (LLMs) in recognizing and classifying objection tactics. If this work measures adequate performance, it may allow more cost effective exploration of objection tactics at scale, particularly across other social media platforms to assess whether tactics differ across different domains (e.g., news versus lifestyle content). Future work should also investigate the interactions with these comments over time [108] and potential impact. Important questions remain as to whether objections draw more replies, or other kinds of attention, from audiences, or whether particular objections stand out in this regard.

This research is not without limitations. The samples of comment replies that we analyzed were composed of videos from U.S. news agencies only, while the comment replies analyzed in Sample 2 were exclusively drawn from CNN. Both samples favored stories relevant to a U.S. audience. We ultimately limited sampling to U.S. sources, then CNN exclusively, because they had consistently more comments than videos posted by foreign news agencies, and these comments were also largely in English where ad hominem attacks could be easily recognized. Future work would benefit from more diverse sampling to build upon the typology presented here. Additionally, MTurk workers were trained to understand and identify distinct objection tactics through online tutorials, confirming construct validity but proving costly with their high rate of attrition between quality check quiz and coding test.

While social media companies have invested considerable time and effort in identifying and removing offensive content, it has not resulted in platforms free of content that people find offensive. The goal of our research was to identify the discursive objection tactics used when humans see and experience a gap in moderation and think a public comment should be stopped. In the process, we developed a typology for classifying types of discursive objection tactics used in public news commentary. Understanding the tactics that people use when objecting to comments is the first step to understanding the role of these behaviors in democratic discourse.

## Supporting information

S1 Table Most common terms in objection replies and non-objection replies. (TIF)

S1 Fig Word cloud of 100 words from our samples with highest Log Odds Ratio. (TIF)

S1 File Example of tutorial. [Example of tutorial & quiz for MTurkers also deposited in OSF and available via this anonymous link: https://osf.io/m2qnk/?view_only=a23a70b0c74b406e97450f53657ccc7d].
(PDF)

S2 Table Words with highest Log Odds Ratio. (TIF)

S2 Fig Differences in the percentage of tactics represented across samples. (TIF)
