# Supplementary figures and images for: Beyond ad hominem attacks: A typology of the discursive tactics used when objecting to news commentary on social media
Source: PLoS One. 2025 Aug 20;20(8):e0328550. doi: 10.1371/journal.pone.0328550 (PMC12367161; doi:10.1371/journal.pone.0328550)

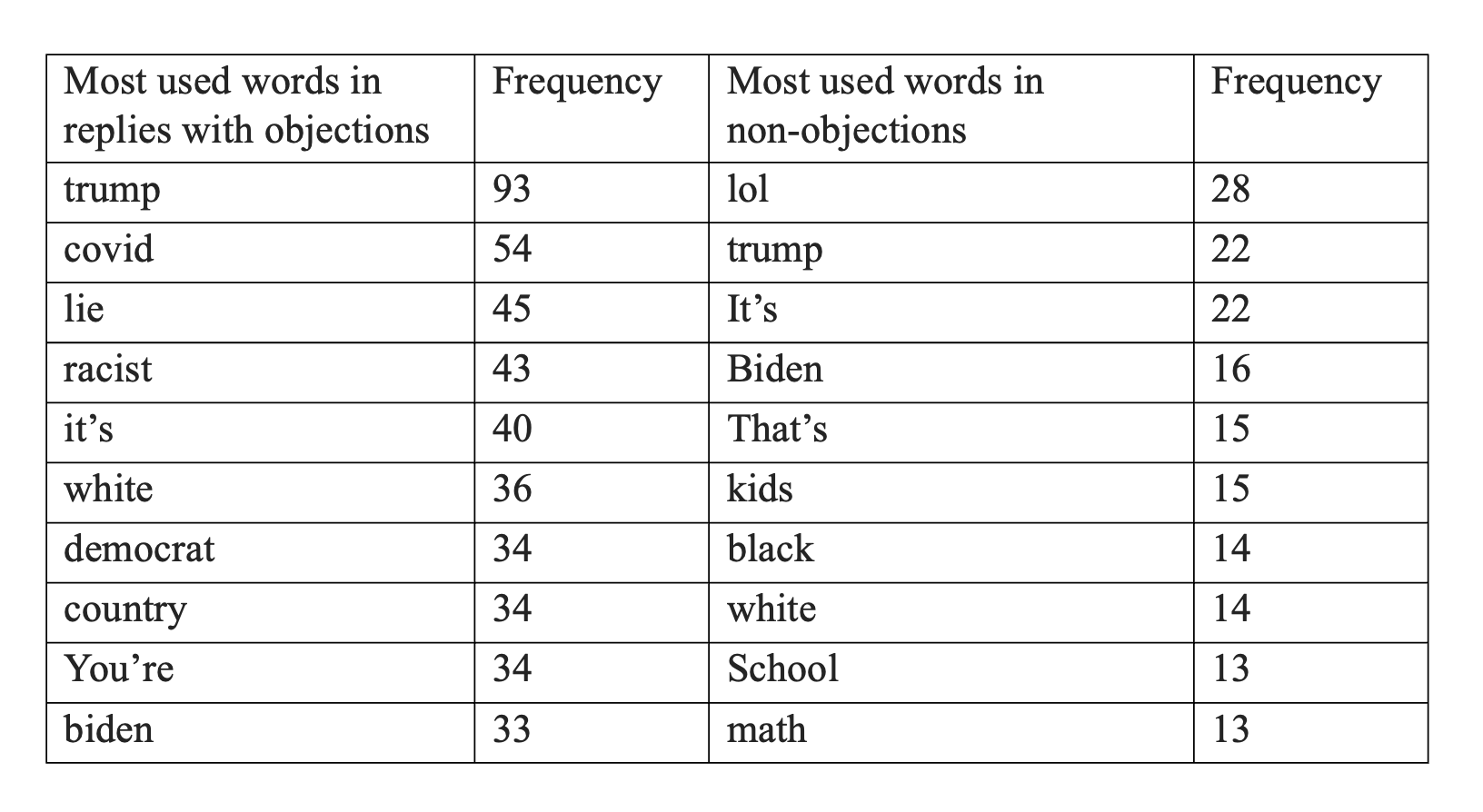

Supplement: S1 Table — (TIF) [file pone.0328550.s001.tif]

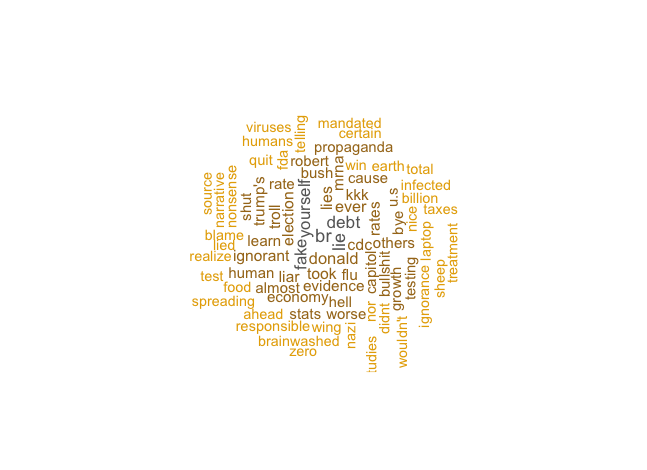

Supplement: S1 Fig — (TIF) [file pone.0328550.s002.tif]

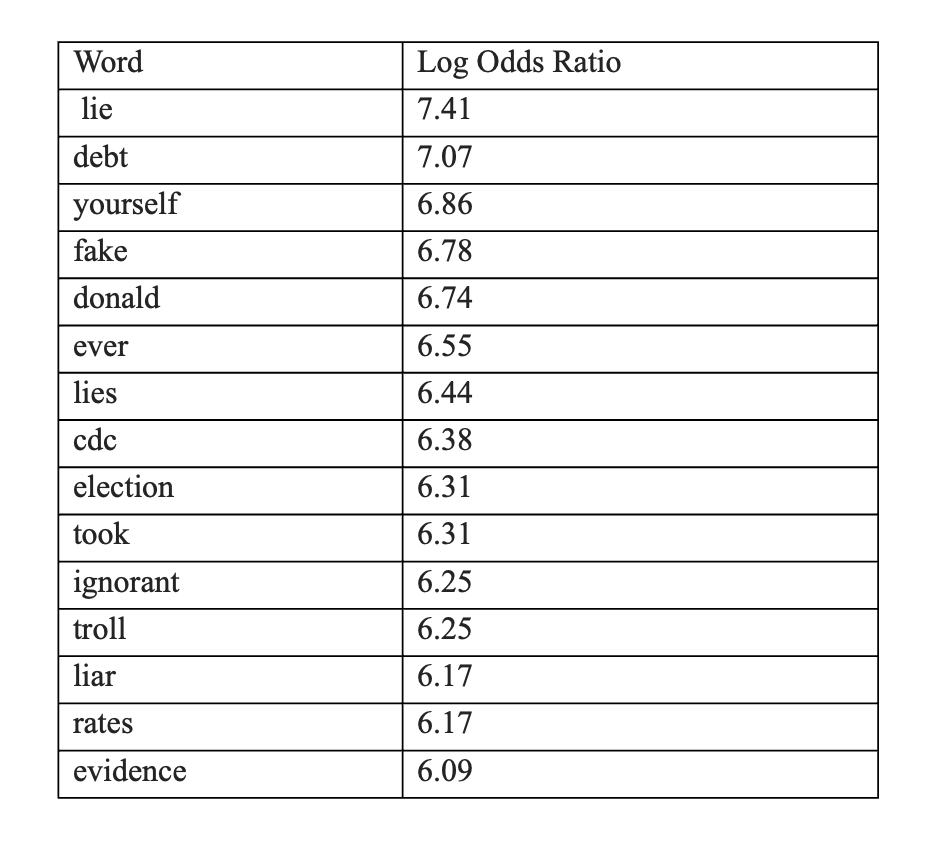

Supplement: S2 Table — (TIF) [file pone.0328550.s004.tif]

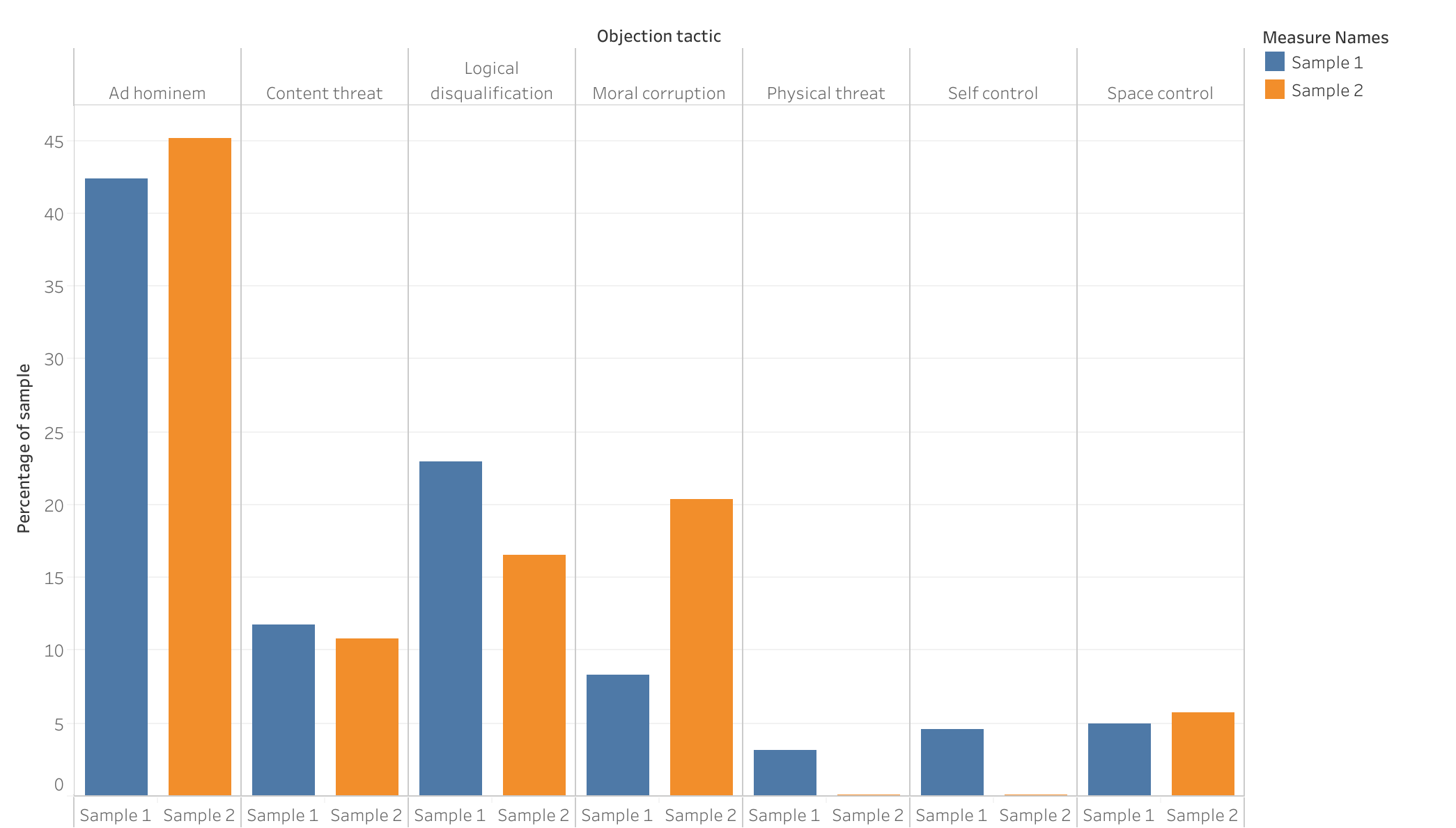

Supplement: S2 Fig — (TIF) [file pone.0328550.s005.tif]
