# Supplementary material for: Beyond ad hominem attacks: A typology of the discursive tactics used when objecting to news commentary on social media
Source: PLoS One. 2025 Aug 20;20(8):e0328550. doi: 10.1371/journal.pone.0328550 (PMC12367161; doi:10.1371/journal.pone.0328550)
Supplement: S1 File — [Example of tutorial & quiz for MTurkers also deposited in OSF and available via this anonymous link: https://osf.io/m2qnk/?view_only=a23a70b0c74b406e97450f53657ccc7d]. (PDF) [file pone.0328550.s003.pdf]

Now it's your turn to try!

Here's what to expect in the quiz:

You will see real comments that were left by users on Twitter or Youtube. These comments were scraped from the public comments section of uploaded videos covering major news stories.

Some comments include **a moral corruption strategy.** Some comments do not use this strategy. Your job is to decide if the comment does or does not use the strategy of **moral corruption.**

You need to answer **5/6** correct to move on to the next section for maximum compensation.

0% 100%

→

Objection Strategy:
Moral corruption

Definition:
Encouraging or chiding a person to be better by appealing to that person's conscience and making it clear that something said was morally corrupt.

Features:
Comments that **implore people to be better** must invoke one of the following:

- morals (like right or wrong; good or evil)
- virtues (like pride, honesty, discipline, etc.)
- humanity (like "future generation" or a general concern for others)
- personal beliefs (like faith, religion, worldview)
- group identity (soldiers, public servants, journalists)

Notes:
These comments should be directed at a specific user, or at a particular group of people represented within the comments section.

General comments that reference the morality or rightness of public figures or generally reference the "future generation" but lack encouragement or chiding to change someone's behavior or beliefs should not be counted here.

1 out of 6 questions

Does the comment below have **Moral Corruption**?

> @User All politicians should be locked up. They are all criminals. Imagine a country so dumb we willfully elect criminals to office who keep lying to us.

○ Yes
○ No

0% 100%

Does the comment below have **Moral Corruption**?

> @User All politicians should be locked up. They are all criminals. Imagine a country so dumb we willfully elect criminals to office who keep lying to us.

No, it does not. This is a general comment about politicians and their supposed corruption but it does not directly try to stop, change or refute the comment of another user.

0% 100%

→

Objection Strategy:
Moral corruption

Definition:
Encouraging or chiding a person to be better by appealing to that person's conscience and making it clear that something said was morally corrupt.

Features:
Comments that **implore people to be better** must invoke one of the following:

- morals (like right or wrong; good or evil)
- virtues (like pride, honesty, discipline, etc.)
- humanity (like “future generation” or a general concern for others)
- personal beliefs (like faith, religion, worldview)
- group identity (soldiers, public servants, journalists)

Notes:
These comments should be directed at a specific user, or at a particular group of people represented within the comments section.

General comments that reference the morality or rightness of public figures or generally reference the "future generation" but lack encouragement or chiding to change someone's behavior or beliefs should not be counted here.

2 out of 6 questions

Does the comment below have **Moral Corruption**?

If you are a veteran then you know you can't say that, be proud and a soldier.

○ Yes
○ No

0% 100%

Does the comment below have **Moral Corruption**?

If you are a veteran then you know you can't say that, be proud and a soldier.

Yes, it does. It references the unspoken expectations and virtues affiliated ("be proud") with being a veteran and soldier.

0% 100%

→

Objection Strategy:
Moral corruption

Definition:
Encouraging or chiding a person to be better by appealing to that person's conscience and making it clear that something said was morally corrupt.

Features:
Comments that **implore people to be better** must invoke one of the following:

- morals (like right or wrong; good or evil)
- virtues (like pride, honesty, discipline, etc.)
- humanity (like “future generation” or a general concern for others)
- personal beliefs (like faith, religion, worldview)
- group identity (soldiers, public servants, journalists)

Notes:
These comments should be directed at a specific user, or at a particular group of people represented within the comments section.

General comments that reference the morality or rightness of public figures or generally reference the "future generation" but lack encouragement or chiding to change someone's behavior or beliefs should not be counted here.

3 out of 6 questions

Does the comment below have **Moral Corruption**?

@User if I were already a millionaire worth 300$ million like Pelosi and her evil 👿 crews are then yes I’d donate my paycheck like our previous president did!

- ◯ Yes
- ◯ No

0% 100%

Does the comment below have **Moral Corruption**?

@User if I were already a millionaire worth 300$ million like Pelosi and her evil 👿 crews are then yes I'd donate my paycheck like our previous president did!

No, it does not. This is a response to a prior question, it seems, but it is a comment about a politician that is not participating in the conversation. The comment attacks the reputation of Nancy Pelosi but not the reputation of someone involved in this conversation.

0% 100%

→

Objection Strategy:
Moral corruption

Definition:
Encouraging or chiding a person to be better by appealing to that person's conscience and making it clear that something said was morally corrupt.

Features:
Comments that **implore people to be better** must invoke one of the following:

- morals (like right or wrong; good or evil)
- virtues (like pride, honesty, discipline, etc.)
- humanity (like “future generation” or a general concern for others)
- personal beliefs (like faith, religion, worldview)
- group identity (soldiers, public servants, journalists)

Notes:
These comments should be directed at a specific user, or at a particular group of people represented within the comments section.

General comments that reference the morality or rightness of public figures or generally reference the "future generation" but lack encouragement or chiding to change someone's behavior or beliefs should not be counted here.

4 out of 6 questions

Does the comment below have **Moral Corruption**?

@User And here lies the problem. It doesn’t matter if you’re afraid of it or not. The pandemic has never been about you. It has always been about the people around you.

- ○ Yes
- ○ No

0% 100%

Does the comment below have **Moral Corruption**?

@User And here lies the problem. It doesn't matter if you're afraid of it or not. The pandemic has never been about you. It has always been about the people around you.

Yes, it does. It appeals to one's obligation to care for others and our collective responsibility to keep the vulnerable safe ("it has always been about the people around you").

0% 100%

→

Objection Strategy:
Moral corruption

Definition:
Encouraging or chiding a person to be better by appealing to that person's conscience and making it clear that something said was morally corrupt.

Features:
Comments that **implore people to be better** must invoke one of the following:

- morals (like right or wrong; good or evil)
- virtues (like pride, honesty, discipline, etc.)
- humanity (like “future generation” or a general concern for others)
- personal beliefs (like faith, religion, worldview)
- group identity (soldiers, public servants, journalists)

Notes:
These comments should be directed at a specific user, or at a particular group of people represented within the comments section.

General comments that reference the morality or rightness of public figures or generally reference the "future generation" but lack encouragement or chiding to change someone's behavior or beliefs should not be counted here.

5 out of 6 questions

Does the comment below have **Moral Corruption**?

@User ... They are so sensitive. They want to give to the poor. Just not with their own money.

- ○ Yes
- ○ No

0% 100%

Does the comment below have **Moral Corruption**?

@User ... They are so sensitive. They want to give to the poor. Just not with their own money.

No, it does not. This comment seems to be ridiculing the act of giving to the poor whereas an appeal to conscience would do the opposite: it would promote the importance of caring for those around us that are less fortunate.

0% 100%

→

Objection Strategy:
Moral corruption

Definition:
Encouraging or chiding a person to be better by appealing to that person's conscience and making it clear that something said was morally corrupt.

Features:
Comments that **implore people to be better** must invoke one of the following:

- morals (like right or wrong; good or evil)
- virtues (like pride, honesty, discipline, etc.)
- humanity (like “future generation” or a general concern for others)
- personal beliefs (like faith, religion, worldview)
- group identity (soldiers, public servants, journalists)

Notes:
These comments should be directed at a specific user, or at a particular group of people represented within the comments section.

General comments that reference the morality or rightness of public figures or generally reference the "future generation" but lack encouragement or chiding to change someone's behavior or beliefs should not be counted here.

6 out of 6 questions

Does the comment below have **Moral Corruption**?

@USER I object to your earlier comment claiming that some of our enlisted personnel enjoyed murdering for the elites. My Cousin was a Navy Dr. who volunteered to take a lower officer's place because that man's wife was pregnant. Kevin was in the first Battalion of Marines who crossed the border in Operation Freedom. They did everything possible to save Civilian Lives. They were ill equipped they had nothing for children casualties so they cut down items to fit kids. Kevin said he knew our guys would be alright they were flown to Italy and Germany, he said what haunted him was given meds to a parent trying to explain how to clean a dressing etc. as they had to move their Unit forward. Kevin held the hands of dying soldiers and civilians, he did not have to but he is a compassionate person..

- ◯ Yes
- ◯ No

Does the comment below have **Moral Corruption**?

@USER I object to your earlier comment claiming that some of our enlisted personnel enjoyed murdering for the elites. My Cousin was a Navy Dr. who volunteered to take a lower officer's place because that man's wife was pregnant. Kevin was in the first Battalion of Marines who crossed the border in Operation Freedom. They did everything possible to save Civilian Lives. They were ill equipped they had nothing for children casualties so they cut down items to fit kids. Kevin said he knew our guys would be alright they were flown to Italy and Germany, he said what haunted him was given meds to a parent trying to explain how to clean a dressing etc. as they had to move their Unit forward. Kevin held the hands of dying soldiers and civilians, he did not have to but he is a compassionate person..

Yes, it does. It explains the honorable actions of a member of the military (retrofitting supplies to accommodate children's needs; holding the hands of dying soldiers and civilians alike) to illustrate their compassionate concern for people in war zones.

0% 100%

→

Your quiz score is **6/6**
Now we will move onto your bonus test for additional compensation!

On the following screen, you will be presented with **8 more comments**. Please answer the question just as you did during the quiz round.

0% 100%

→
